# O Raro Padrão de Pré-Excitação Alternante: Fenômeno Genuinamente Benigno?

**DOI:** 10.36660/abc.20220864

**Published:** 2023-01-24

**Authors:** Mirella Facin, Nelson Samesima

**Affiliations:** 1 Hospital das Clínicas Faculdade de Medicina Universidade de São Paulo São Paulo SP Brasil Instituto do Coração (InCor) - Hospital das Clínicas HCFMUSP - Faculdade de Medicina da Universidade de São Paulo, São Paulo, SP – Brasil

**Keywords:** Nó Atrioventricular, Síndrome de Wolff-Parkinson-White, Prevalência, Grau de risco

O sistema de condução é um conjunto de células miocárdicas especializadas, organizadas para gerar e transmitir os estímulos elétricos, levando às contrações coordenadas e sequenciais de cada ciclo cardíaco.^1^ Uma espessa camada de tecido fibroso, o *annulus fibrosus* , isola quase totalmente o miocárdio atrial do ventricular, exceto na região do nodo atrioventricular (AV) e sistema His-Purkinje que, no coração normal, constitui o único caminho elétrico entre as câmaras superiores e inferiores.^1 , 2^

Em alguns indivíduos, entretanto, feixes musculares que conectam diretamente átrios e ventrículos, remanescentes de etapas anteriores de desenvolvimento cardíaco, perduram após o nascimento.^2^ A presença de vias atrioventriculares adicionais ou acessórias (VA) permite que o estímulo elétrico contorne o nodo AV, resultando na ativação precoce dos ventrículos ou pré-excitação. Vias acessórias podem cruzar o sulco atrioventricular em qualquer ponto de justaposição entre o miocárdio atrial e ventricular. A maioria delas tem propriedades de condução bidirecional e não decremental, e pode servir como braço de circuitos ortodrômicos/antidrômicos reentrantes, ou como via rápida para a transmissão do estímulo elétrico durante outras taquicardias supraventriculares, tais como a fibrilação atrial (FA).^3^

A condução anterógrada do ritmo sinusal através de uma via acessória modifica a sequência temporal e espacial da ativação cardíaca, levando a um padrão eletrocardiográfico (ECG) típico composto de 1) intervalo PR curto, 2) empastamento inicial do QRS ou onda “delta”, e 3) prolongamento do QRS, em níveis variados de expressão.^4 , 5^ Descrito pela primeira vez em 1930,^6^o padrão eletrocardiográfico de Wolff-Parkinson-White (WPW), ou pré-excitação ventricular, é relativamente raro, com prevalência reportada de 1-3:1.000 indivíduos.^7 - 10^ Sintomas arrítmicos ocorrem em um quinto dos pacientes com pré-excitação e definem a síndrome de WPW.^5^

Palpitações, tontura, síncope e dor torácica são apresentações clínicas comuns e na maioria das vezes causadas por taquicardias atrioventriculares reentrantes (TAVR). Possivelmente relacionada à instabilidade elétrica inerente às vias acessórias, a fibrilação atrial incide em 20-30% dos pacientes.^5^ A condução AV rápida de arritmias atriais pode degenerar em fibrilação ventricular e morte súbita cardíaca (MSC), a manifestação mais temida da síndrome WPW.^5^ Embora eventos de MSC sejam consideravelmente mais frequentes em pacientes com sintomas (risco vitalício estimado em 4%),^11^ esse risco não é nulo nos portadores assintomáticos, atingindo quase 0,13% ao ano em uma metanálise de 1.869 pacientes.^12^ Episódios arrítmicos são ainda mais frequentes em pacientes com pré-excitação ventricular que realizam atividade física extenuante. Embora marcadores não invasivos possam contribuir na identificação de vias acessórias de baixo risco, diretrizes recentes sugerem postergar o treinamento físico desses pacientes até estratificação invasiva adequada.^5 , 13^ Idade precoce, indutibilidade de taquicardia AV reentrante durante estimulação elétrica programada, múltiplas vias acessórias e a demonstração da capacidade da via acessórias em admitir condução rápida aos ventrículos – menor intervalo R-R pré-excitado durante FA (SPERRI) ≤ 250 ms, ou um curto período refratário anterógrado efetivo (PRE) ≤ 250 ms – foram associados ao aumento do risco.^5 , 13^ Reconhecer o padrão eletrocardiográfico de WPW é, portanto, obrigatório, mas nem sempre tarefa simples.

A evidência de pré-excitação ao ECG depende do tempo necessário para que o ritmo sinusal alcance o nó AV e a via acessória, da velocidade de condução em cada via atrioventricular e de seu PRE.^13^ Muitos fatores, como localização da via acessória, tônus autonômico, distúrbios metabólicos e drogas, podem afetar as variáveis mencionadas, tornando dinâmica a expressão do padrão de WPW. Ondas “delta” podem ser marcantes, sutis, imperceptíveis, ou mesmo desaparecer de tempos em tempos. Deveras, a pré-excitação intermitente é relatada em até 15% dos casos.^14^ Um representante curioso deste fenômeno é a pré-excitação alternante, na qual complexos QRS alargados e com onda delta se alternam com QRS normais, batimento a batimento, no mesmo traçado eletrocardiográfico, um importante diagnóstico diferencial de outras causas de alternância do QRS, como bloqueio de ramo intermitente, bigeminismo atrial e ventricular. Dados sobre essa apresentação particular do padrão WPW eram escassos e anedóticos, disponíveis em alguns relatos de caso. No entanto, Lim et al. abordaram consistentemente a prevalência de pré-excitação alternante nos registros médicos pré-participação de mais de 125 mil recrutas militares do sexo masculino.^15^ O padrão WPW ocorreu em 184 (0,147%) indivíduos, frequência semelhante à relatada por extensos estudos epidemiológicos. Dentre os portadores do padrão WPW, a pré-excitação alternante foi reportada em apenas em quatro (2,2%) pacientes – dois desses desenvolveram sintomas relacionados a TAVR. Embora a perda intermitente da pré-excitação tenha sido historicamente associada a vias acessórias de baixo risco, estudos recentes em sujeitos sintomáticos e assintomáticos demonstraram que mais de um quinto dos pacientes com padrão WPW intermitente apresentaram vias acessórias com PRE <250 ms.^15^ Infelizmente, Lim et al. não puderam fornecer informações sobre estratificação de risco dessa forma tão particular de pré-excitação, devido ao pequeno número de pacientes com padrão WPW alternante, à ausência de informações eletrofisiológicas invasivas e ao limitado período de seguimento de seu trabalho.^15^ São necessários mais estudos para confirmar a prevalência relatada de pré-excitação alternante e avaliar seu impacto prognóstico.
