# Supplementary material for: O Raro Padrão de Pré-Excitação Alternante: Fenômeno Genuinamente Benigno?
Source: Arq Bras Cardiol. 2023 Jan 24;120(1):e20220864. [Article in Portuguese] doi: 10.36660/abc.20220864 (PMC10389101; doi:10.36660/abc.20220864)
Supplement: Translation

# Translation: The Rare Alternans Pre-Excitation Pattern: Is It a Genuinely Benign Phenomenon?

**Authors:** Mirella Facin, Nelson Samesima

**Affiliations:** 1 Brazil Instituto do Coração (InCor) - Hospital das Clínicas HCFMUSP - Faculdade de Medicina da Universidade de São Paulo, São Paulo, SP – Brazil

**Keywords:** Atrioventricular Node, Wolff-Parkinson-White Syndrome, Prevalence, Risk Grade

The conduction system is a specialized myocardial cell arrangement that neatly generates and transmits electrical stimuli, leading to sequentially coordinated contractions in each cardiac cycle.^1^A thick layer of fibrous tissue, the annulus fibrosus, almost entirely insulates the atrial from the ventricular myocardia, except in the region of the atrioventricular (AV) node and His-Purkinje system, which, in normal hearts, stands as the single electric pathway between the upper and lower chambers.^1 , 2^

In some individuals, however, threads of myocardial tissue directly connecting atria to ventricles remain from previous developmental stages and endure after childbirth.^2^The presence of additional atrioventricular conduits or accessory pathways (AP) allows the electrical stimuli to bypass the AV node, resulting in earlier activation of the ventricles or pre-excitation. Bypass tracts may cross the atrioventricular groove wherever atrial and ventricular myocardium are juxtaposed. Most are capable of bidirectional and non-decremental conduction and may serve as a limb to reentrant orthodromic/antidromic circuits or fast lanes for impulse transmission during other supraventricular tachycardias, such as atrial fibrillation (AF).^3^

The antegrade sinus rhythm conduction through an accessory pathway modifies the temporal and spatial sequence of cardiac activation, leading to a typical electrocardiographic (ECG) pattern composed of 1) a short PR interval, 2) initial QRS slurring or “delta” wave, and 3) QRS enlargement, in various degrees of expression.^4 , 5^First described in 1930,^6^the so-called pre-excitation or Wolff-Parkinson-White (WPW) pattern is relatively rare, accounting for 1-3:1000 individuals.^7 - 10^Arrhythmic symptoms occur in one-fifth of the patients with pre-excitation and define the WPW syndrome.^5^

Palpitations, light-headedness, syncope, and thoracic pain are common clinical presentations and often relate to reentrant atrioventricular tachycardias. Possibly triggered by the electrical instability inherent to APs, atrial fibrillation arises in 20-30% of patients.^5^The rapid AV conduction of atrial arrhythmias may degenerate into ventricular fibrillation, leading to sudden cardiac death (SCD), WPW syndrome’s most feared manifestation.^5^The risk of SCD is considerably high for patients with symptoms, approaching 4% over a lifetime;^11^yet, it is not null in asymptomatic carriers, reaching almost 0.13% per year in a meta-analysis comprising 1869 patients.^12^Pre-excitation-related arrhythmic events are even more frequent in patients undergoing strenuous physical activity. Albeit non-invasive markers could add to the identification of low-risk APs, recent guidelines suggest postponing exercise training in those patients until proper invasive risk stratification.^5 , 13^Younger age, inducibility of AV-reentrant tachycardia during programmed electrical stimulation, numerous accessory pathways, and demonstration of the bypass tract capability to admit rapid conduction to ventricles – i.e., shortest pre-excited RR interval during AF (SPERRI) of ≤ 250 ms at baseline or an AP short antegrade effective refractory period (ERP) of ≤ 250 ms – were associated to increased risk.^5 , 13^Recognizing the WPW ECG pattern is hence mandatory but not always straightforward.

The extent to which pre-excitation is evident on ECG depends on the time taken for the sinus rhythm to reach the AV node and the accessory pathway, on the conduction velocity in each atrioventricular trail, and their ERP.^13^Many factors, such as AP location, autonomic tone, metabolic disturbances, and drugs, can affect the abovementioned variables and make the WPW pattern expression dynamic. “Delta” waves can be marked or subtle, maybe unnoticed, even disappearing from time to time. Indeed, intermittent pre-excitation is reported in up to 15% of cases.^14^A curious representative of this phenomenon is the alternans pre-excitation, in which enlarged delta-waved QRS complexes alternate with normal ones, on a beat-to-beat basis, in the same ECG trace, an important differential diagnosis to other causes of QRS alternans, such as intermittent branch block, atrial and ventricular bigeminy. Data on this particular WPW pattern presentation was scarce and anecdotal, available in a few case reports. However, Lim et al. consistently addressed alternans pre-excitation prevalence by reviewing the pre-participation medical files from over 125 thousand male military recruits.^15^WPW pattern occurred in 184 (0.147%) individuals, a frequency similar to that of extensive epidemiologic studies. Beneath the cases of WPW pattern, alternans pre-excitation was rare, occurring in only four (2.2%) patients – half of them further developed AVRT-related symptoms. Although the intermittent loss of pre-excitation has historically been associated with low-risk AP, recent studies with both symptomatic and asymptomatic subjects demonstrated that over one-fifth of patients with intermittent WPW pattern presented AP ERP <250 ms.^15^Unfortunately, Lim et al. could not provide information on risk stratification due to the small number of patients presenting alternans pre-excitation, the absence of invasive electrophysiologic information, and the limited follow-up period in their study.^15^More studies are needed to confirm the reported prevalence of alternans pre-excitation and evaluate its impact on patient prognosis.
